# The different contributions of the eight prefrontal cortex subregions to reactive responses after unpredictable slip perturbations and vibrotactile cueing

**DOI:** 10.3389/fnhum.2023.1236065

**Published:** 2023-09-07

**Authors:** Beom-Chan Lee, Jongkwan Choi, Jooeun Ahn, Bernard J. Martin

**Affiliations:** ^1^Department of Health and Human Performance, Center for Neuromotor and Biomechanics Research, University of Houston, Houston, TX, United States; ^2^Institute of Sport Science, Seoul National University, Seoul, Republic of Korea; ^3^OBELAB Inc., Seoul, South Korea; ^4^Department of Physical Education, Seoul National University, Seoul, Republic of Korea; ^5^Department of Industrial and Operations Engineering, University of Michigan, Ann Arbor, MI, United States

**Keywords:** slip perturbation, pre-warning cue, prefrontal cortex, fNIRS, kinematics, step response time

## Abstract

**Introduction:**

Recent advancements in functional near-infrared spectroscopy technology have offered a portable, wireless, wearable solution to measure the activity of the prefrontal cortex (PFC) in the human neuroscience field. This study is the first to validate the different contributions made by the PFC's eight subregions in healthy young adults to the reactive recovery responses following treadmill-induced unpredictable slip perturbations and vibrotactile cueing (i.e., precues).

**Methods:**

Our fall-inducing technology platform equipped with a split-belt treadmill provided unpredictable slip perturbations to healthy young adults while walking at their self-selected walking speed. A portable, wireless, wearable, and multi-channel (48 channels) functional near-infrared spectroscopy system evaluated the activity of PFC's eight subregions [i.e., right and left dorsolateral prefrontal cortex (DLPFC), ventrolateral prefrontal cortex (VLPFC), frontopolar prefrontal cortex (FPFC), and orbitofrontal cortex (OFC)] as quantified by oxyhemoglobin and deoxyhemoglobin concentrations. A motion capture system and two force plates beneath the split-belt treadmill were used to quantify participants' kinematic and kinetic behavior. All participants completed 6 trials: 2 consecutive trials without vibrotactile cueing and with a slip perturbation (control trials); 3 trials with vibrotactile cueing [2 trials with the slip perturbation (cueing trial) and 1 trial without the slip perturbation (catch trial)], and 1 trial without vibrotactile cueing and with a slip perturbation (post-control trial). The PFC subregions' activity and kinematic behavior were assessed during the three periods (i.e., standing, walking, and recovery periods).

**Results:**

Compared to the walkers' standing and walking periods, recovery periods showed significantly higher and lower levels of oxyhemoglobin and deoxyhemoglobin concentrations, respectively, in the right and left DLPFC, VLPFC, and FPFC, regardless of the presence of vibrotactile cueing. However, there was no significant difference in the right and left OFC between the three periods. Kinematic analyses confirmed that vibrotactile cueing significantly improved reactive recovery responses without requiring more involvement by the PFC subregions, which suggests that the sum of attentional resources is similar in cued and non-cued motor responses.

**Discussion:**

The results could inform the design of wearable technologies that alert their users to the risks of falling and assist with the development of new gait perturbation paradigms that prompt reactive responses.

## 1. Introduction

Falls are a major global health concern in all populations, particularly the elderly (Talbot et al., 2005; World Health Organization, 2021). Falls and fall-related injuries affect the quality of life by increasing an individual's anxiety and fear of falling while performing daily activities (Zaloshnja et al., 2005; 2008). Regardless of age, slips and trips account for the majority of unintentional falls while walking (Talbot et al., 2005; Heijnen and Rietdyk, 2016). Although multiple factors (e.g., physical, neurological, physiological, and environmental) can also increase the risk of falling, the ability to respond quickly to an unexpected slip, trip, stumble, etc. is critical for successful recovery (Talbot et al., 2005; Heijnen and Rietdyk, 2016; Raina et al., 2016).

Previous studies have demonstrated that regular physical activity or exercise reduces the risk of falling by improving muscle strength and flexibility, enhancing balance control and endurance, promoting coordination of body segments, and facilitating the integration of sensory information (Gschwind et al., 2013; Vieira et al., 2016). Because physical activity or exercise cannot address the full range of unpredictable trips and slips, a number of fall-inducing technologies have been developed to improve the recovery responses of balanced-constrained individuals during walking (see Mccrum et al., 2017 for a review). The technologies are based on the concept that motor learning, training, and adaptation can facilitate balance and locomotor recovery. Multiple studies have also demonstrated that fall-inducing technologies incorporating mechanical obstacles (e.g., Schillings et al., 2000; Pavol et al., 2001; Bieryla et al., 2007), cables/pulleys (e.g., Mansfield et al., 2010; Shirota et al., 2011), programmable treadmills (e.g., Lee et al., 2016, 2017a,b), and gait perturbation paradigms can improve recovery responses (i.e., compensatory motor responses).

Recovery responses from falls on slippery surfaces have motivated the development of shoe-based technologies with sensors that detect ground-level obstacles (Zhang et al., 2011; Otis and Menelas, 2012; Lin et al., 2017). Miniature cameras, ultrasonic, and/or infrared sensors differentiate the ground's physical characteristics (e.g., deformable vs. non-deformable) (Otis and Menelas, 2012), and sensory cueing modalities (visual, audible, and/or vibrotactile) alert the user. The obvious advantage of vibrotactile cueing is that it does not interfere with a user's own visual and auditory cues while walking (Kondo et al., 2017). Notably, vibrotactile cueing about an impending trip perturbation has been found to be more efficient than learning from repeated exposure to unpredictable trips (Lee et al., 2016, 2017a,b).

Recently, we confirmed that the activity of the eight subregions of the prefrontal cortex (PFC) increased during balance recovery following unpredictable gait perturbations (Lee et al., 2020). Similarly, some neuroimaging studies have confirmed that balance recovery in response to surface perturbations while standing requires more attentional resources in the PFC responsible for executive functions (e.g., responses to novel events, action updating, planning, monitoring, and integrating self-generated information) to organize the sequence of purposeful motor actions (Mihara et al., 2008; Basso Moro et al., 2014; Fujimoto et al., 2014). Other studies have suggested that processing augmented sensory biofeedback or cueing requires more attentional resources (Lin et al., 2015; Krecisz and Kuczynski, 2018).

Motivated by the lack of studies on PFC subregion-specific contributions to motor responses after unpredictable slip perturbations and vibrotactile cueing, our study evaluates their activity by using functional near-infrared spectroscopy (fNIRS), a neuroimaging technique that offers several advantages over functional magnetic resonance imaging, electroencephalography, etc. (e.g., ease-of-use, unobtrusiveness, relatively low cost, and relatively good spatial and temporal resolution) (see Scholkmann et al., 2014; Herold et al., 2017, 2018 for review). Our study has the following three objectives: (1) to evaluate the specific contributions of PFC to reactive recovery responses following unpredictable slip perturbations while walking on a treadmill; (2) to investigate how PFC subregions process vibrotactile cueing about impending unpredictable slip perturbations; and (3) to analyze the relationship between PFC activity and kinematic behavior during reactive recovery responses.

## 2. Methods

### 2.1. Experimental apparatus

**Figure 1:**
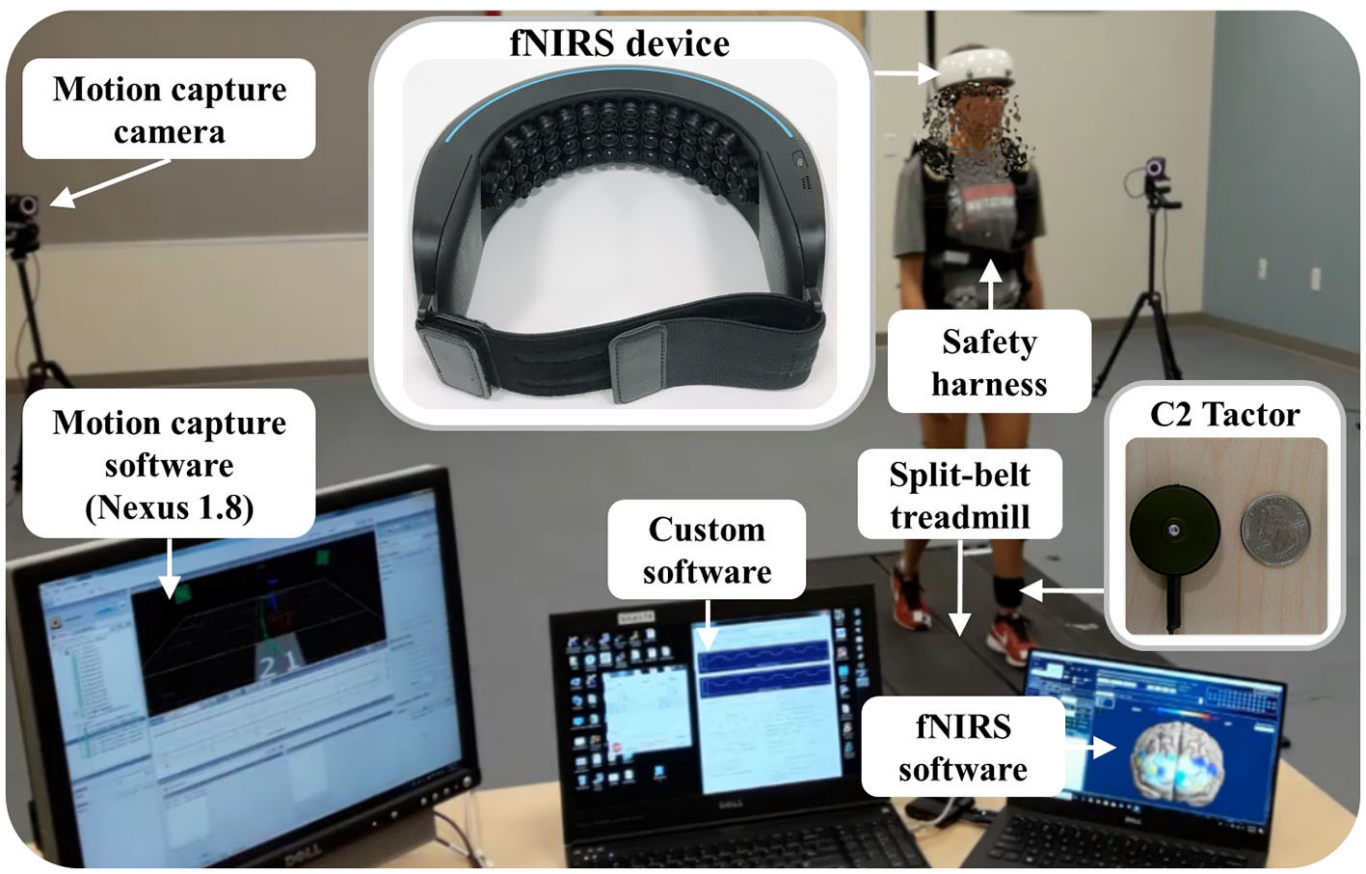
Experimental apparatus and associated software.

Figure 1 depicts our experimental apparatus: a fall-inducing technology platform, an fNIRS device (NIRSIT, OBELAB Inc., Seoul, S. Korea), a 12-camera motion capture system (VICON, Vicon Motion Systems Ltd., Oxford, UK), and a customized control unit for vibrotactile cueing (C2 tactor, Engineering Acoustics Inc., Casselberry, FL, USA). The fNIRS device is a portable, wireless, wearable, and multi-channel (48 channels) brain imaging system. The technology platform incorporates a programmable split-belt treadmill equipped with two force plates under each belt (Bertec Corporation, Columbus, OH, USA) and our custom-developed software that runs the real-time gait phase detection algorithm, controls each belt to provide unpredictable slip perturbations, and generates vibrotactile cueing (Lee et al., 2016, 2017a,b). Accelerating one of the belts in either the anterior or posterior direction (i.e., the perturbation occurs at foot level) generates the slip perturbations.

The fNIRS device covers the forehead completely without hair interference. It has 24 laser sources and 32 photodetectors separated by a unit distance of 1.5 cm; each laser source emits 780 nm and 850 nm wavelengths with a power of <1 mW. Hence, the fNIRS device measures the optical power of the wavelength pair for 48 channels with the most common 3-cm source-detector separation (Herold et al., 2017). Manufacturer specifications indicate that the penetration depth of the laser is ~2 cm from the skin, the weight is 550 g, and the sampling rate is 8.138 Hz. It wirelessly transmits the measured optical power to the laptop recording the data.

The 12-camera motion capture system uses 35 reflective passive markers to measure the body kinematics typically used in gait studies. Its Nexus 1.8 software continuously samples the markers' positions and ground reaction forces (GRFs) from the two force plates of the split-belt treadmill at a rate of 100 Hz.

The customized control unit for vibrotactile cueing (C2 tactor) is a commonly used linear actuator with a moving contactor. The contactor, shielded within a passive housing, oscillates perpendicularly to the skin. Our custom software wirelessly sends a command signal to the control unit that generates the 250 Hz sinusoidal signal driving the C2 tactor. The peak-to-peak displacement amplitude of vibration is approximately 200 μm at the selected frequency (Lee et al., 2013), which primarily stimulates cutaneous receptors. Therefore, a significant role for reflex contribution and muscle proprioception in modifying motor responses is ruled out (Lee et al., 2012, 2013).

Our custom software, fNIRS software, and Nexus 1.8 are synchronized to start and stop recording simultaneously (i.e., our custom software sends a start and stop signal to Nexus 1.8 via a data acquisition device (NI-6211, National Instruments Co., Austin, TX, USA) and to the fNIRS software via an Ethernet connection). It also sends event signals associated with the experimental protocols to both Nexus 1.8 and fNIRS software (see Section 2.3). The measured communication delay of the synchronization and event signals is <1 ms.

### 2.2. Participants

The 10 recruited healthy young adults (5 females and 5 males; age: 23.9 ± 3.5 years; stature: 171.5 ± 7.1 cm; mass: 66.2 ± 13.2 kg) were naïve to the purpose of the study and had never participated in studies of gait perturbations. Exclusion criteria included self-reported neurological disorders (e.g., stroke and Parkinson's disease), musculoskeletal dysfunctions, peripheral sensory diseases (e.g., peripheral neuropathy and type 2 diabetes), pregnancy, left-footedness, and a body mass index (BMI) >30 kg/m^2^ [BMIs over 30 may affect gaits (Corbeil et al., 2001; Himes and Reynolds, 2012; Wu et al., 2012)].

The University of Houston Institutional Review Boards approved the study protocol, which accorded with the Helsinki Declaration. Each participant reviewed and signed the informed consent prior to the study.

### 2.3. Experimental protocol

Participants were instrumented with the 35 markers on the head, neck, shoulders, arms, trunk, knees, and feet. All wore an adjustable safety harness. All were instrumented with the C2 tactor on the skin over the left lower leg around the lateral fibularis longus area, a location chosen based on our previous finding of a consistent and immediate reactive response to gait perturbations regardless of the vibrotactile cueing's location (upper arm, trunk, and lower leg) (Lee et al., 2016, 2017a). Each participant self-selected a walking speed by adjusting the treadmill's speed. The average self-selected walking speed was 0.8 ± 0.2 m/s.

All participants completed 6 trials: 2 consecutive trials without vibrotactile cueing and with a slip perturbation (control trials); 3 trials with vibrotactile cueing [2 trials with the slip perturbation (cueing trial) and 1 trial without the slip perturbation (catch trial)]; and 1 trial without vibrotactile cueing and with a slip perturbation (post-control trial). The three trials with vibrotactile cueing were randomized. Based on our previous studies, we determined that six trials would eliminate the potential for learning effects (Lee et al., 2016, 2017a,b). The fNIRS device was calibrated for each participant with the fNIRS software to prevent signal saturation due to biological factors (e.g., skin and hair color, skin and skull thickness) (Kim et al., 2018). In particular, the output power of each laser source was controlled under 1 mW to balance the signal intensity of the 780-nm and 850-nm wavelengths for each channel. We adjusted the gain of each photodetector to maximize the signal intensity without reaching saturation for each channel.

Each trial consisted of a standing period (15 s quiet standing), a walking period (steady state walking at the self-selected speed), and a post-perturbation period (from the perturbation onset to the trial's end). The custom software increased the speed of the treadmill's two belts between the standing and walking periods and decreased it after the post-perturbation period at a rate of 0.2 m/s^2^. During the control, cueing, and post-control trials, a randomized perturbation was applied to the left foot loading phase [10% of the gait cycle, corresponding to the initial double-limb support (Lee et al., 2016, 2017a)] between the 31st and 40th step by accelerating the left belt of the treadmill in the anterior direction at a rate of 10 m/s^2^, to induce a backward slip. The accelerated belt returned to the pre-perturbation speed with the first heel strike of the right foot (i.e., the first step response of the non-slipping foot) because stepping is a common recovery response (Mcilroy and Maki, 1993; Jensen et al., 2001; Maki and Mcilroy, 2006). All trials ended 10 steps after the perturbation by considering the number of required steps to return to normal walking (Lee et al., 2016, 2017a) and the temporal delay (i.e., latency) between the cortical activity and the hemodynamic response of ~4 to 7 s (Herold et al., 2017). Normal walking resumed within three or four steps after the perturbation, corresponding to 4.2 ± 0.9 s. The average time of the post-perturbation period was 11.5 ± 1.1 s. During the cueing and catch trials, vibrotactile cueing was applied to the left lower leg 250 ms prior to the perturbation and was stopped with the first heel strike of the right foot (Lee et al., 2016, 2017a). A lead time for vibrotactile cueing (i.e., 250 ms prior to the perturbation) was chosen based on our previous finding of a consistent reactive response to gait perturbations regardless of the vibrotactile cueing's lead time (250 ms vs. 500 ms) (Lee et al., 2016, 2017a). The participants received no information about the onset of slip perturbations, the presence of vibrotactile cueing, or how they should respond. However, they were informed that vibrotactile cueing indicates the imminence of a slip perturbation.

To minimize head movements and side-to-side walking variations, participants walked on the split-belt treadmill at their self-selected walking speed while fixing their gaze on an “X” mark placed ~4.5 m ahead at eye level (Lee et al., 2016, 2017a). Consecutive trials were separated by a 20 s rest period so participants could briefly relax the torso and upper and lower extremities.

### 2.4. Data and statistical analyses

The Nexus 1.8 ran the Plug-in-Gait model to compute the whole-body kinematics. MATLAB (The MathWorks, Natick, MA, USA) processed the optical power, whole-body kinematics, and GRFs.

**Figure 2:**
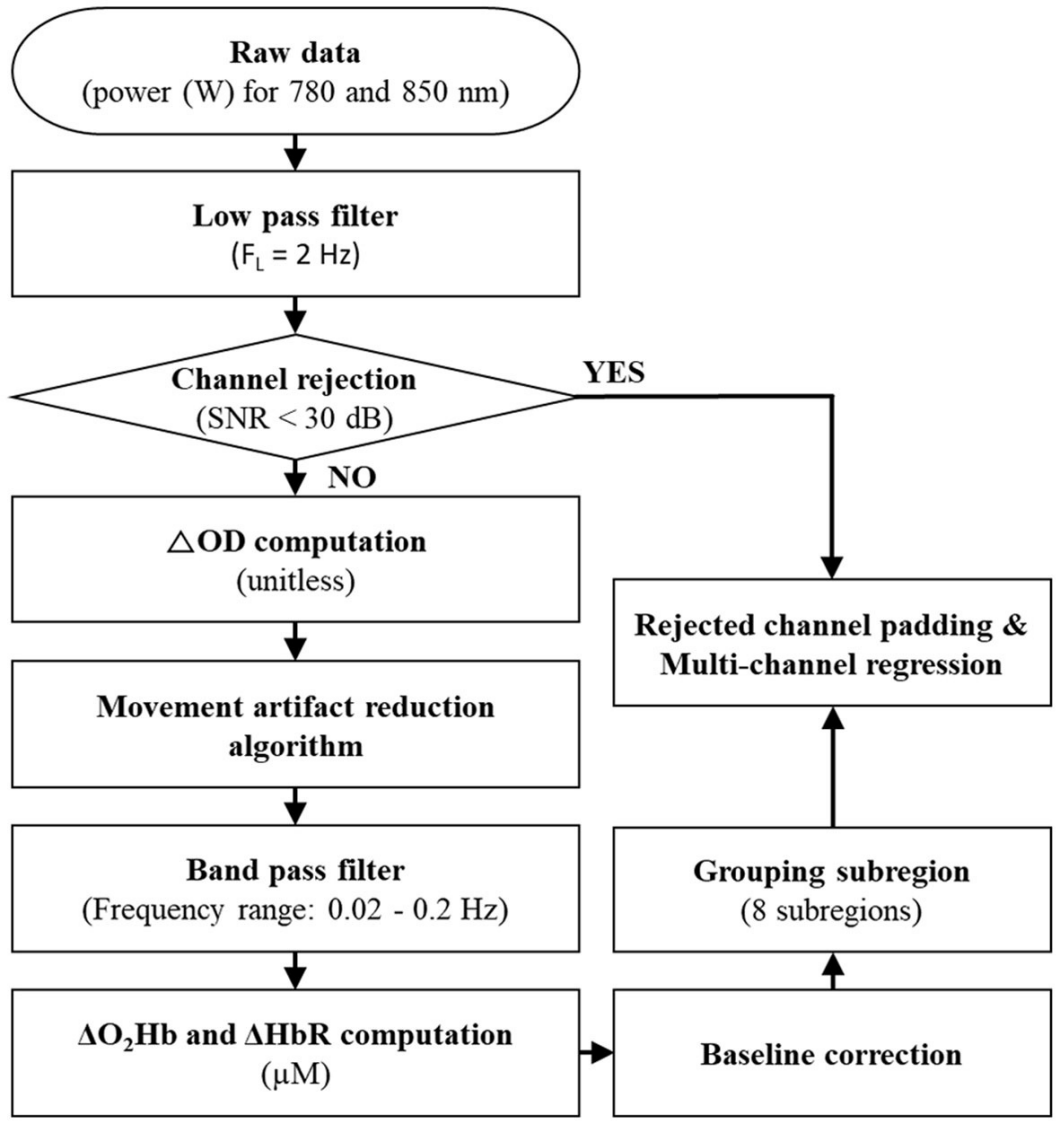
Signal processing steps for fNIRS data. SNR, ΔOD, ΔO_2_Hb, and ΔHbR indicate a signal-to-noise ratio, changes in optical density, oxyhemoglobin concentrations, and deoxyhemoglobin concentrations, respectively.

Since an increase in PFC activity correlates with an increase in oxyhemoglobin concentrations (ΔO_2_Hb) and a concomitant decrease in deoxyhemoglobin concentrations (ΔHbR) (Villringer and Chance, 1997), both ΔO_2_Hb and ΔHbR for all 48 channels were quantified based on the series of signal processing techniques depicted in Figure 2.

A low-pass filter with a 2 Hz cutoff frequency was applied to the fNIRS's raw data (i.e., the optical power of the used wavelength pair) to eliminate high-frequency instrumental and environmental noises based on the power spectral density analysis of the raw data. The application of a signal-to-noise ratio (SNR) of <30 dB rejected unreliable or distorted channels; SNR was defined as the ratio of a mean to a standard deviation of the fNIRS's filtered data for the baseline first 5 s of the recorded period (corresponding to one-third of the quiet standing period). The SNR threshold of 30 dB was chosen because it effectively extracted reliable ΔO_2_Hb and ΔHbR in the presence of noise signals of <0.1 Hz caused by blood circulation (Choi et al., 2016). The average rejected channels were 2.0 ± 2.9 in each trial.

After the channel rejection process, changes in optical density (ΔOD) relative to the baseline period for the 780-nm and 850-nm wavelengths were computed for the accepted channels (Delpy et al., 1988). The fNIRS neuroimaging technique is sensitive to motion artifacts (see Vitorio et al., 2017 for a review). Therefore, the movement artifact reduction algorithm (Scholkmann et al., 2010), one of the most successful algorithms as confirmed by Cooper et al. (2012), was applied to the computed ΔOD. Next, the computed ΔOD was filtered by a bandpass filter with a frequency range between 0.02 Hz and 0.2 Hz to remove equipment noises, respiration, heart pulsation, and other irrelevant physiological effects (Cui et al., 2014). After applying the bandpass filter, ΔO_2_Hb and ΔHbR were computed by the modified Beer–Lambert law (Delpy et al., 1988). A baseline correction (i.e., normalization) was performed by subtracting the average of ΔO_2_Hb and ΔHHb corresponding to the baseline period from the computed ΔO_2_Hb and ΔHHb.

**Figure 3:**
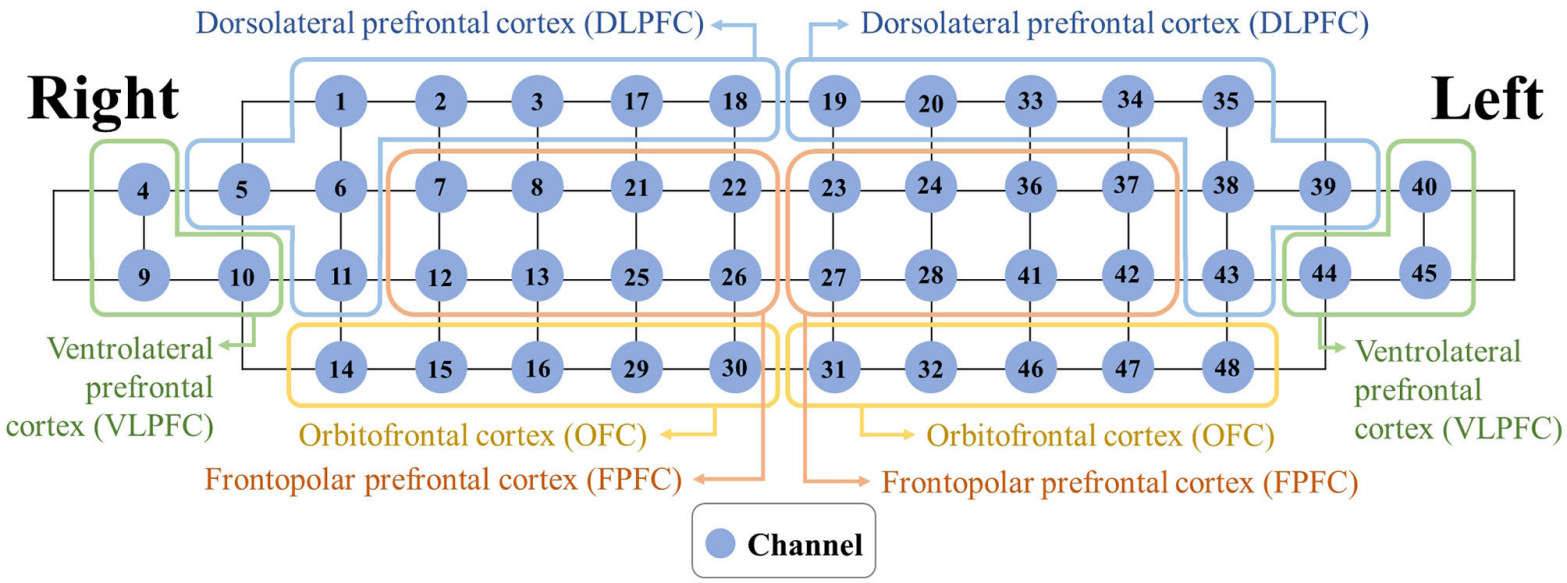
Mapping between each subregion and associated channels.

Figure 3 depicts the grouping of the 48 channels into the eight subregions [right and left dorsolateral prefrontal cortex (DLPFC), ventrolateral prefrontal cortex (VLPFC), frontopolar prefrontal cortex (FPFC), and orbitofrontal cortex (OFC)].

The Montreal Neurological Institute transformation was conducted by individual location coordinates in four positions [nasion, right pre-auricular, left pre-auricular, and central zero from the 10-20 system (Homan et al., 1987)] based on the probabilistic registration method after affine transformation, and the International Consortium for Brain Mapping 152 template (Singh et al., 2005). Rejected channels were padded by the average of the accepted channels within each subregion. To reduce possible signal contamination caused by extra-brain artifacts (e.g., scalp blood flow and systemic blood flow), a multi-channel regression method was applied to each subregion (Pfeifer et al., 2018). Finally, the activity of the eight subregions was quantified by ΔO_2_Hb_AVG_ and ΔHbR_AVG_).

**Figure 4:**
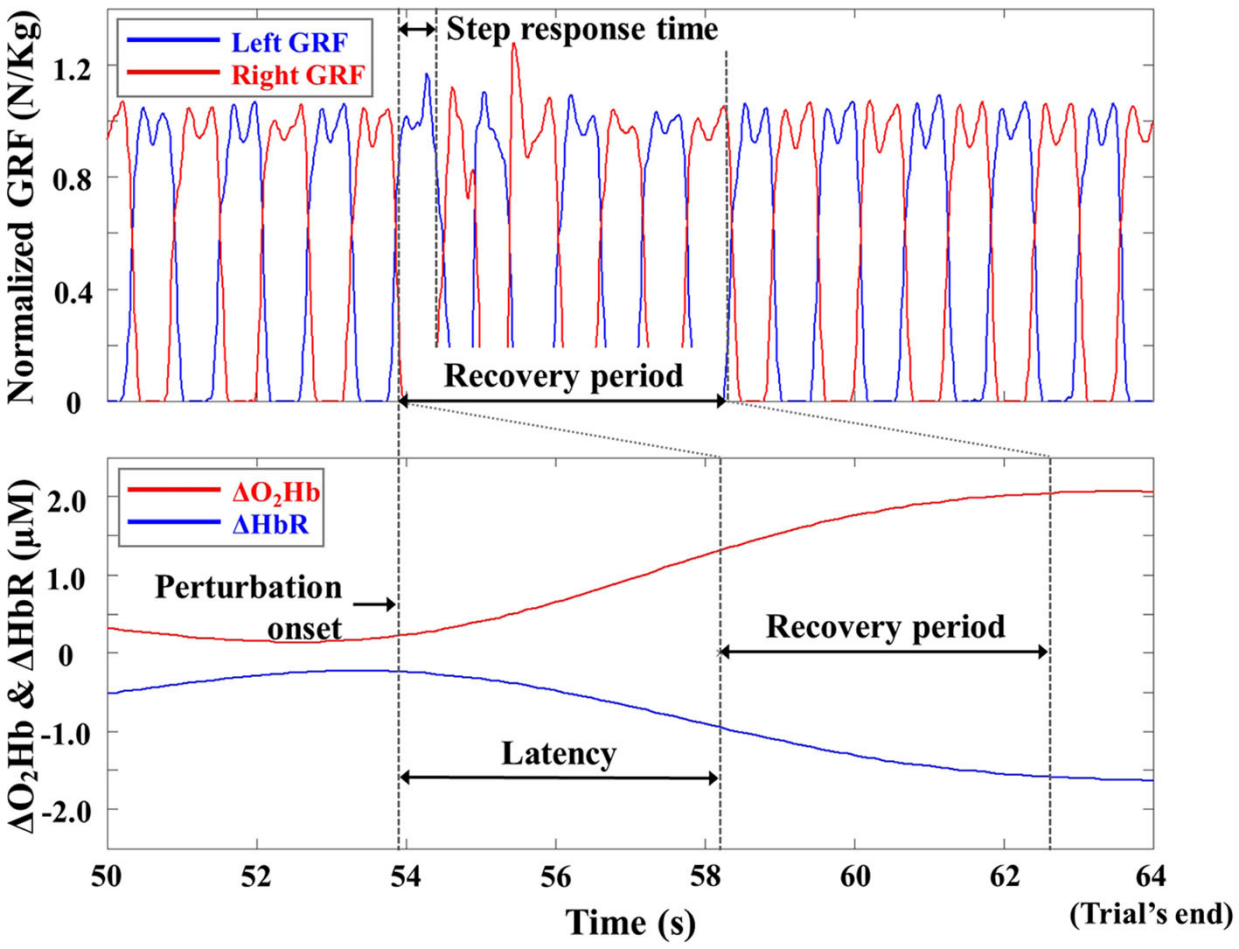
Representative ground reaction force (GRF), ΔO_2_Hb, and ΔHbR profiles from one trial of one participant. The GRFs are normalized to the body weight of the participant for illustrative purposes.

Figure 4 depicts one participant's recovery period for the computed body kinematics and recorded GRFs, defined from the perturbation onset to the instant of return to normal walking (Lee et al., 2016, 2017a). To perform latency-dependent analysis, the recovery period for ΔO_2_Hb_AVG_ and ΔHbR_AVG_ was shifted by a response latency value. To compute a response latency value, the onset of PFC activity was detected by employing the moving average convergence divergence (MACD) filter (Durantin et al., 2014). The MACD filter is a digital passband filter based on the principle of exponential moving averages. The onset of PFC activity was detected when MACD-filtered fNIRS signals crossed a threshold (i.e., ΔO_2_Hb = 0.5 μM). To simplify data analysis, the computed response latency values of bilateral DLPFC, VLPFC, and FPFC were averaged for each trial, since the OFC activity did not change (see Section 3).

The computed body kinematics and recorded GRFs were low-pass filtered with a 10 Hz cutoff frequency to remove unwanted noises (Winter, 2009). Previous studies have shown that reactive recovery responses following slip perturbations can be quantified by variables from the kinematic data (e.g., Owings et al., 2001; Bhatt et al., 2013; Lee et al., 2016, 2017a,b), primarily those controlling the trunk, arms, whole-body center of mass (COM), and feet. Therefore, trunk angular dispersion (AD), trunk range of motion (ROM), COM ROM, minimum COM position, and step response time of the right foot (i.e., non-slip foot) were selected. The trunk AD, trunk ROM, COM ROM, and minimum COM position were computed as an average during the standing, walking, and recovery periods (Lee et al., 2016, 2017a). Trunk AD represented the first standard deviation of trunk angular displacement and trunk ROM represented the range of motion in degrees between the flexion and extension maxima with respect to the vertical direction. Since the participants differed in height, COM positions were normalized to the initial COM position relative to the vertical direction. COM ROM indicated its range of motion in cm between the maximum elevation and depression along the vertical direction with respect to the horizontal reference. The computed trunk AD and ROM corresponded to components in the sagittal plane, and the computed COM ROM and minimum COM position corresponded to those in the transverse plane, where whole-body movements largely predominated due to a backward slip (Lee et al., 2017b). The step response time from the start of the slip perturbation to the first heel strike of the non-slip foot (i.e., right foot) was defined using recorded GRFs (Lee et al., 2016, 2017a).

The six variables (i.e., ΔO_2_Hb_AVG_, ΔHbR_AVG_, trunk AD, trunk ROM, COM ROM, and minimum COM position) were computed as a function of the period for each trial. The two variables, response latency and step response time, were computed only during the recovery period for each trial.

All statistical analyses used SPSS (IBM Corp., Armonk, NY, USA) for the ΔO_2_Hb_AVG_, ΔHbR_AVG_, latency, trunk AD, trunk ROM, COM ROM, minimum COM, and step response time. The Shapiro–Wilk test confirmed that the outcome measures were distributed normally. Statistical analyses consisted of an initial analysis and a main analysis. In the initial analysis, a repeated measure analysis of variance (RMANOVA) assessed the effects of gender and trial repetitions (i.e., 2 control trials and 2 trials with vibrotactile cueing) for all dependent metrics. Since the RMANOVA showed no significant effects of gender or trial repetitions, the eight outcome measures for the 2 control trials and the 2 trials with vibrotactile cueing were averaged as a function of the period for each participant. In the main analysis, a two-way ANOVA determined the main effect of the four trial conditions (i.e., control, cueing, catch, and post-control), the three periods (standing, walking, and recovery), and the interactions (condition × period) for ΔO_2_Hb_AVG_, ΔHbR_AVG_, trunk AD, trunk ROM, COM ROM, and minimum COM position. A one-way ANOVA determined the main effect of the vibrotactile cueing on the response latency and step response time variables only during the recovery period for each trial. An *F*-test tested the hypotheses for the main effects of the independent variables and their interactions. A *post-hoc* analysis using the Šidák method determined the factors influencing the main and interaction effects. The Šidák method is a statistical procedure that has been extensively used for pairwise comparisons. It is an extension of the Bonferroni correction and is intended to limit the family-wise error rate when conducting multiple comparisons simultaneously (Abdi, 2007). For all statistical analyses, the level of significance was defined at a *p*-value < 0.05.

## 3. Results

### 3.1. Prefrontal cortex activity

Tables 1, 2 summarize the results of the statistical analyses for ΔO_2_Hb_AVG_ and ΔHbR_AVG_. The two-way ANOVA applied to ΔO_2_Hb_AVG_ and ΔHbR_AVG_ showed significant main effects of the trial condition, period, and trial condition × period interaction for the right and left DLPFC, VLPFC, and FPFC. There were insignificant main effects of the trial condition, period, and trial condition × period interaction for the right and left OPC.

**Table 1:** Statistical analysis results of average oxyhemoglobin concentrations (ΔO_2_Hb_AVG_) as a function of the eight subregions for trial condition (C), period (P), and interaction (C × P).

**Subregion**	**Effects**	**DF**	***F*-value**	***p*-value**
Right DLPFC	C	3, 108	3.371	0.021^*^
	P	2, 108	253.407	< 0.0001^***^
	C × P	6, 108	2.343	0.036^*^
Left DLPFC	C	3, 108	2.866	0.040^*^
	P	2, 108	193.226	< 0.0001^***^
	C × P	6, 108	2.323	0.038^*^
Right VLPFC	C	3, 108	2.746	0.047^*^
	P	2, 108	160.981	< 0.0001^***^
	C × P	6, 108	2.918	0.011^*^
Left VLPFC	C	3, 108	2.980	0.035^*^
	P	2, 108	164.756	< 0.0001^***^
	C × P	6, 108	2.692	0.018^*^
Right FPFC	C	3, 108	4.396	0.006^**^
	P	2, 108	224.051	< 0.0001^***^
	C × P	6, 108	3.839	0.002^**^
Left FPFC	C	3, 108	3.817	0.012^*^
	P	2, 108	232.302	< 0.0001^***^
	C × P	6, 108	3.050	0.009^**^
Right OPC	C	3, 108	0.079	0.971
	P	2, 108	0.195	0.823
	C × P	6, 108	0.107	0.995
Left OPC	C	3, 108	0.113	0.952
	P	2, 108	0.102	0.903
	C × P	6, 108	0.074	0.998

* *p* < 0.05,

** *p* < 0.01, and

*** *p* < 0.0001.

**Table 2:** Statistical analysis results of average oxyhemoglobin concentrations (ΔHbR_AVG_) as a function of the eight subregions for trial condition (C), period (P), and interaction (C × P).

**Subregion**	**Effects**	**DF**	***F*-value**	***p*-value**
Right DLPFC	C	3, 108	4.590	0.005^**^
	P	2, 108	312.473	< 0.0001^***^
	C × P	6, 108	3.152	0.007^**^
Left DLPFC	C	3, 108	2.714	0.048^*^
	P	2, 108	179.189	< 0.0001^***^
	C × P	6, 108	2.822	0.014^*^
Right VLPFC	C	3, 108	3.794	0.012^*^
	P	2, 108	278.740	< 0.0001^***^
	C × P	6, 108	2.941	0.011^*^
Left VLPFC	C	3, 108	3.101	0.030^*^
	P	2, 108	273.129	< 0.0001^***^
	C × P	6, 108	2.632	0.020^*^
Right FPFC	C	3, 108	4.034	0.009^**^
	P	2, 108	316.659	< 0.0001^***^
	C × P	6, 108	4.695	< 0.0001^***^
Left FPFC	C	3, 108	3.717	0.014^*^
	P	2, 108	291.272	< 0.0001^***^
	C × P	6, 108	2.590	0.022^*^
Right OPC	C	3, 108	0.204	0.894
	P	2, 108	0.026	0.974
	C × P	6, 108	0.150	0.989
Left OPC	C	3, 108	0.028	0.994
	P	2, 108	0.048	0.953
	C × P	6, 108	0.114	0.999

* *p* < 0.05,

** *p* < 0.01, and

*** *p* < 0.0001.

**Figure 5:**
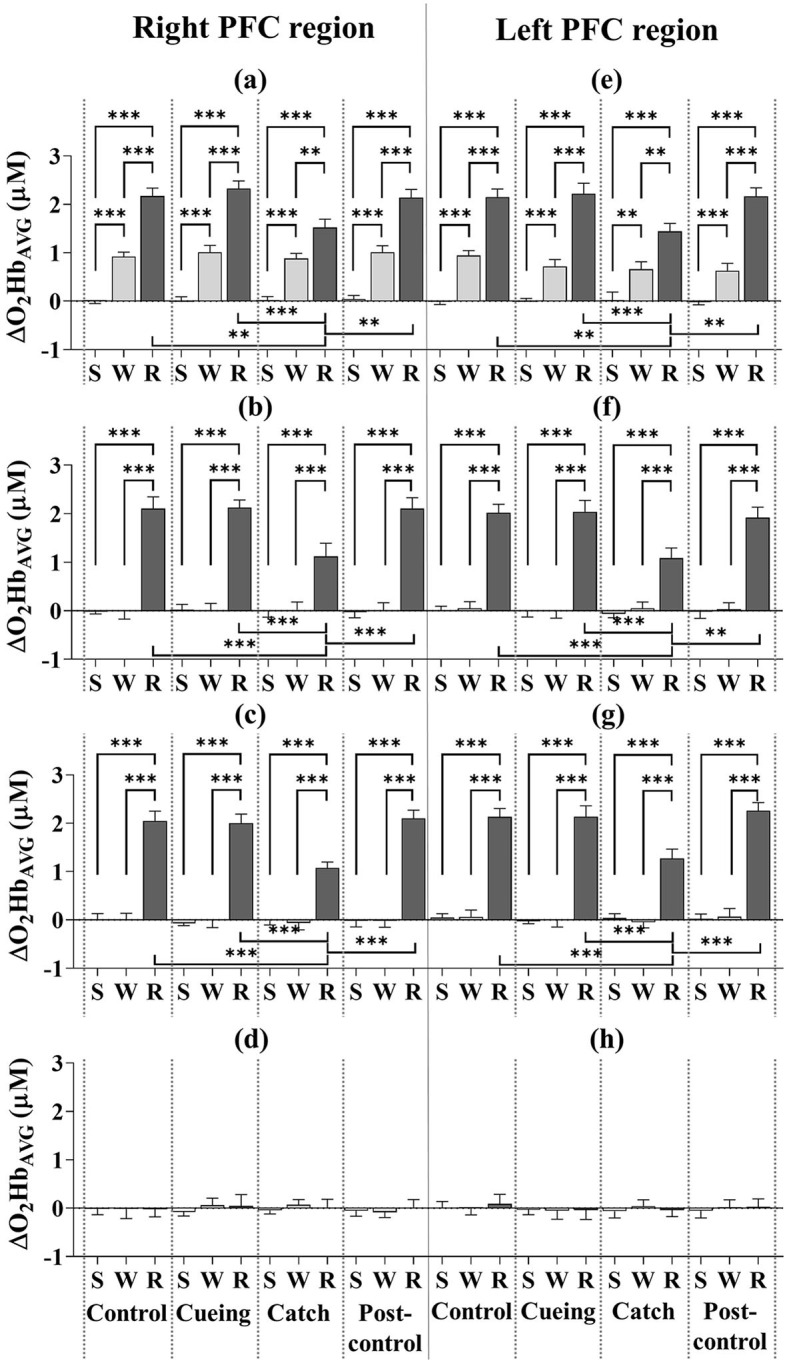
Average oxyhemoglobin concentrations (ΔO_2_Hb_AVG_) as a function of PFC subregion, trial condition, and period across all participants. **(a)** Right DLPFC (dorsolateral prefrontal cortex). **(b)** Right VLPFC (ventrolateral prefrontal cortex). **(c)** Right FPFC (frontopolar prefrontal cortex). **(d)** Right OFC (orbitofrontal cortex). **(e)** Left DLPFC (dorsolateral prefrontal cortex). **(f)** Left VLPFC (ventrolateral prefrontal cortex). **(g)** Left FPFC (frontopolar prefrontal cortex). **(h)** Left OFC (orbitofrontal cortex). S, W, and R indicate standing, walking, and recovery periods, respectively. Error bars indicate the standard error of the corresponding mean (**p < 0.01 and ***p < 0.0001).

Figure 5 depicts the ΔO_2_Hb_AVG_ for the eight subregions as a function of the trial condition and period, including the statistical significance resulting from the *post-hoc* multiple comparisons. The *post-hoc* analysis showed that the ΔO_2_Hb_AVG_ of the right and left DLPFC, VLPFC, and FPFC was significantly higher in magnitude during the recovery period than the standing and walking periods, regardless of trial condition. For only the recovery period, the increased level of the ΔO_2_Hb_AVG_ for the right and left DLPFC, VLPFC, and FPFC was significantly lower for the catch trial than for the control, cueing, and post-control trials. The *post-hoc* multiple comparisons between the control, cueing, and post-control trials were insignificant. The *post-hoc* analysis showed that the ΔO_2_Hb_AVG_ of the right and left DLPFC was significantly higher in magnitude during the walking period than the standing period, regardless of trial condition. For the right and left VLPFC and FPFC, the *post-hoc* multiple comparisons between the standing and walking periods were insignificant, regardless of trial condition. Furthermore, a significant increase in the magnitude of the right and left DLPFC, VLPFC, and FPFC activities during the recovery period compared to the standing and walking periods resulted in a significant interaction (condition × period) effect.

**Figure 6:**
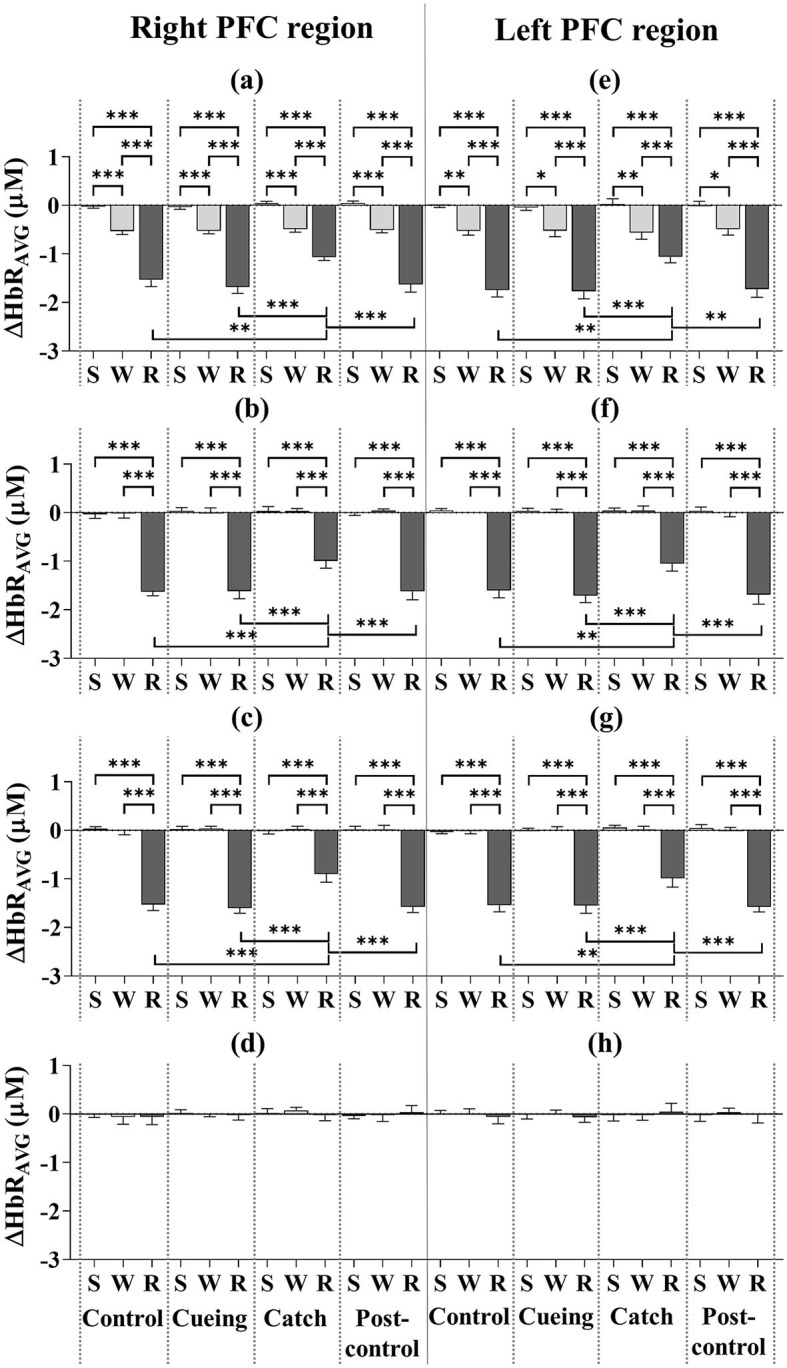
Average deoxyhemoglobin concentrations (ΔHbR_AVG_) as a function of PFC subregion, trial condition, and period across all participants. **(a)** Right DLPFC (dorsolateral prefrontal cortex). **(b)** Right VLPFC (ventrolateral prefrontal cortex). **(c)** Right FPFC (frontopolar prefrontal cortex). **(d)** Right OFC (orbitofrontal cortex). **(e)** Left DLPFC (dorsolateral prefrontal cortex). **(f)** Left VLPFC (ventrolateral prefrontal cortex). **(g)** Left FPFC (frontopolar prefrontal cortex). **(h)** Left OFC (orbitofrontal cortex). S, W, and R indicate standing, walking, and recovery periods, respectively. Error bars indicate the standard error of the corresponding mean (*p < 0.05, **p < 0.01, and ***p < 0.0001).

Figure 6 shows the ΔHbR_AVG_ for the eight subregions as a function of the trial condition and period, including the statistical significance resulting from the *post-hoc* multiple comparisons. The *post-hoc* analysis showed that the ΔHbR_AVG_ of the right and left DLPFC, VLPFC, and FPFC was significantly lower in magnitude during the recovery period than the standing and walking periods, regardless of the trial condition. For only the recovery period, the decreased level of the ΔHbR_AVG_ for the right and left DLPFC, VLPFC, and FPFC was significantly lower for the catch trial compared to the control, cueing, and post-control trials. The *post-hoc* multiple comparisons between the control, cueing, and post-control trials were insignificant. The *post-hoc* analysis showed that the ΔHbR_AVG_ of the right and left DLPFC was significantly lower in magnitude during the walking period than the standing period, regardless of the trial condition. For the right and left VLPFC and FPFC, the *post-hoc* multiple comparisons between the standing and walking periods were insignificant regardless of the trial condition. Furthermore, a significant decrease in the magnitude of the right and left DLPFC, VLPFC, and FPFC activities during the recovery period compared to the standing and walking periods resulted in a significant interaction (condition × period) effect.

**Figure 7:**
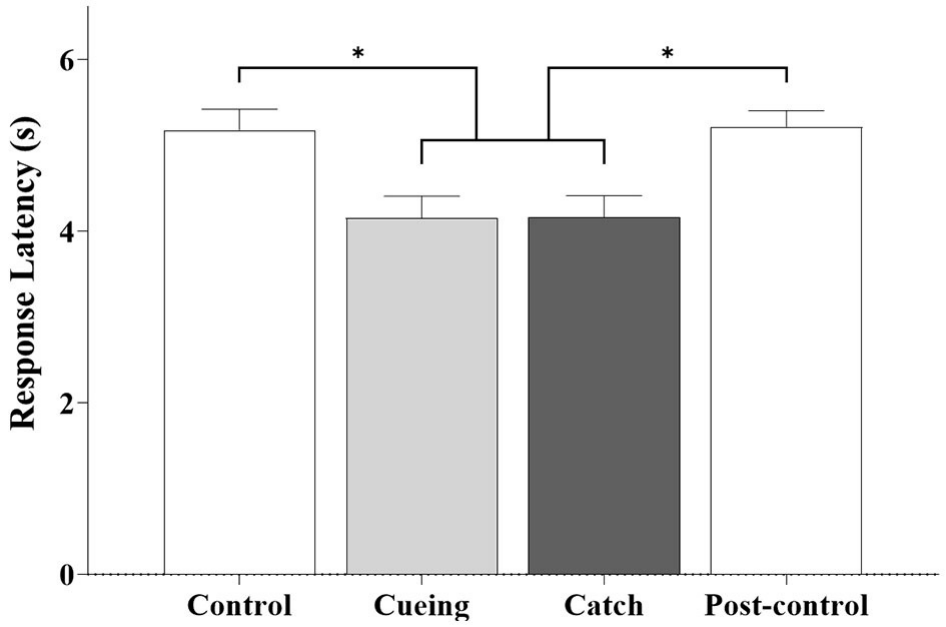
Average response latency as a function of trial condition across all participants. Error bars indicate the standard error of the corresponding mean (*p < 0.05).

Figure 7 shows the average response latency as a function of the trial condition, including the statistical significance resulting from the *post-hoc* multiple comparisons. The one-way ANOVA applied to the response latency showed a significant main effect of the trial condition [*F* (3.36) = 6.540, *p* = 0.001]. The *post-hoc* analysis showed that response latency was significantly shorter for the cueing and catch trials than for the control and post-control trials. The *post-hoc* multiple comparisons between the cueing and catch trials and the control and post-control trials were insignificant.

### 3.2. Kinematics

Table 3 summarizes the results of the statistical analysis for the trunk AD, trunk ROM, COM ROM, and minimum COM position. The two-way ANOVA showed significant main effects of the trial condition, period, and trial condition × period interaction for the four kinematic variables.

**Table 3:** Statistical analysis results of kinematic variables as a function of the eight subregions for trial condition (C), period (P), and interaction (C × P).

**Variable**	**Effects**	**DF**	***F*-value**	***p*-value**
Trunk AD	C	3, 108	5.055	0.003^**^
	P	2, 108	78.719	< 0.0001^***^
	C × P	6, 108	5.811	< 0.0001^***^
Trunk ROM	C	3, 108	6.802	< 0.0001^***^
	P	2, 108	96.950	< 0.0001^***^
	C × P	6, 108	7.220	< 0.0001^***^
COM ROM	C	3, 108	10.551	< 0.0001^***^
	P	2, 108	318.137	< 0.0001^***^
	C × P	6, 108	11.522	< 0.0001^***^
Minimum COM position	C	3, 108	7.167	< 0.0001^***^
	P	2, 108	160.523	< 0.0001^***^
	C × P	6, 108	3.696	< 0.0001^***^

** *p* < 0.01 and

*** *p* < 0.0001.

**Figure 8:**
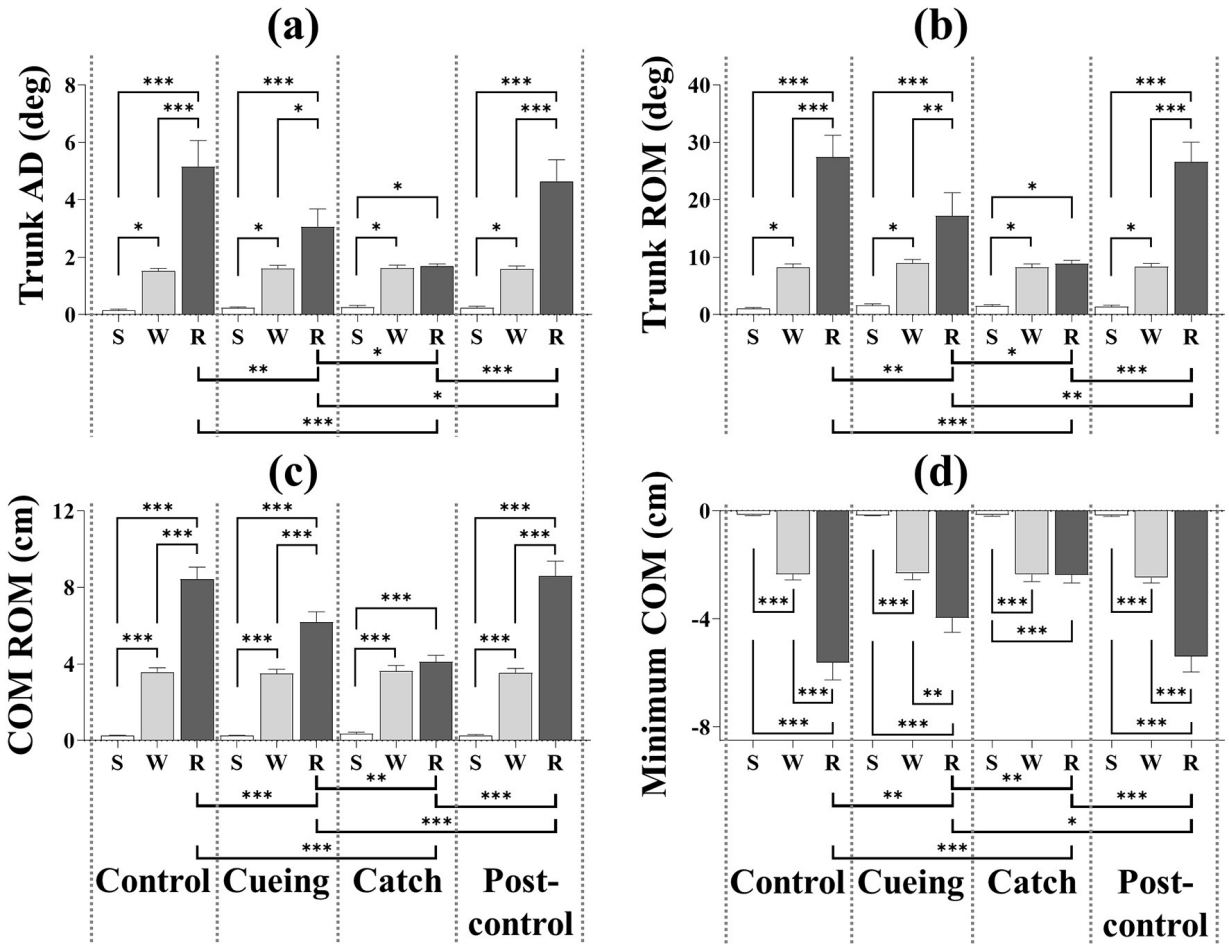
Average kinematic variables as a function of trial condition and period across all participants. **(a)** Trunk AD. **(b)** Trunk ROM. **(c)** COM ROM. **(d)** Minimum COM position. S, W, and R indicate standing, walking, and recovery periods, respectively. Error bars indicate the standard error of the corresponding mean (*p < 0.05, **p < 0.01, and***p < 0.0001).

Figure 8 depicts the results of the four kinematic variables as a function of the trial condition and period, including the statistical significance resulting from the *post-hoc* multiple comparisons. The *post-hoc* analysis showed that trunk AD, trunk ROM, and COM ROM were significantly higher during the recovery period than the standing and walking periods for the control, cueing, and post-control trials. The *post-hoc* analysis for the same trials also showed that the minimum COM position was significantly lower during the recovery period than for the standing and walking periods. Furthermore, a significant increase in the trunk AD, trunk ROM, and COM ROM and a significant decrease in the minimum COM position during the recovery period compared to the standing and walking periods resulted in a significant interaction (condition × period) effect.

For only the catch trial, the *post-hoc* multiple comparisons of trunk AD, trunk ROM, COM ROM, and minimum COM position showed insignificant differences between the walking and recovery periods. The *post-hoc* analysis also showed that trunk AD, trunk ROM, and COM ROM were significantly higher during the walking period than the standing period regardless of the trial condition, and that the minimum COM position was significantly lower during the walking period than the standing period regardless of the trial condition.

For only the recovery period, the *post-hoc* analysis showed that the catch trial had the smallest trunk AD, trunk ROM, and COM ROM and the largest minimum COM position compared to the other trials. The same analysis also showed that the cueing trial had the second smallest trunk AD, trunk ROM, and COM ROM and the second lowest minimum COM position compared to the other trials. The *post-hoc* multiple comparisons of trunk AD, trunk ROM, COM ROM, and minimum COM position between the control and post-control trials were insignificant, which indicated no learning effect by repetition.

**Figure 9:**
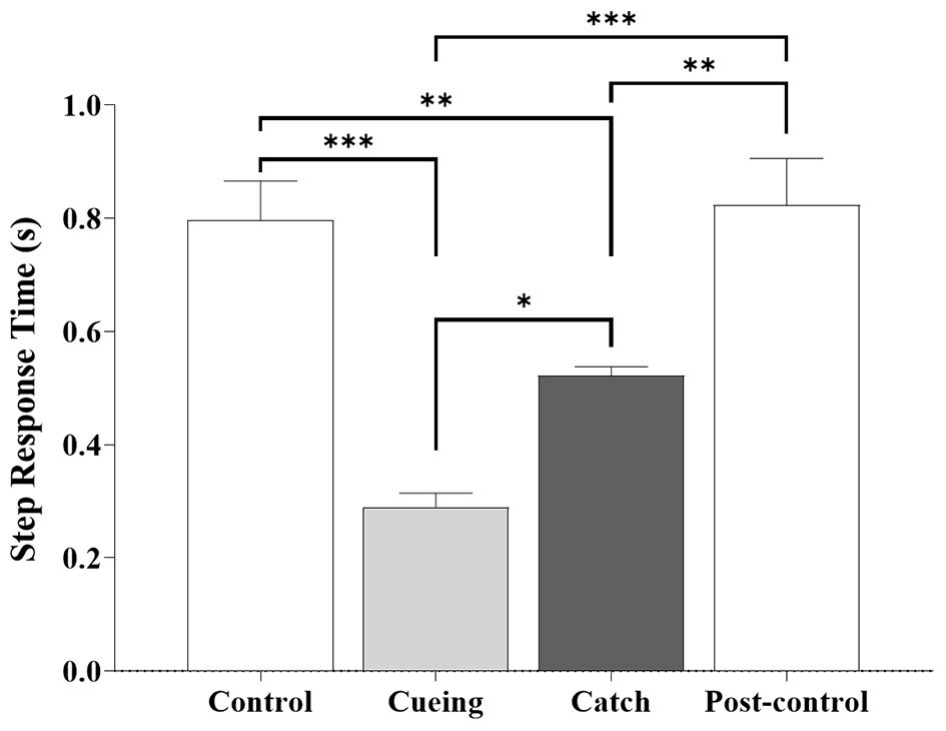
Average step response time as a function of trial condition across all participants. Error bars indicate the standard error of the corresponding mean (*p < 0.05, **p < 0.01, and ***p < 0.0001).

The one-way ANOVA applied to the step response time showed a significant main effect of the trial condition [*F* (3.36) = 12.470, *p* < 0.0001]. Figure 9 shows the average step response time as a function of the trial condition, including the statistical significance resulting from the *post-hoc* multiple comparisons.

The *post-hoc* analysis showed that the step response time during the cueing and catch trials was significantly shorter compared to the control and post-control trials. The same analysis showed that the cueing and catch trials had the smallest and second smallest step response times, respectively. The *post-hoc* multiple comparisons between the control and post-control trials were insignificant, which indicated no learning effect from repetition. The step response time (0.52 ± 0.05 s) of the catch trial was similar to the average right step time (0.54 ± 0.05 s) during the walking period of the catch trial.

## 4. Discussion

This study found differences in the contributions of the PFC's eight subregions to the reactive recovery responses following unpredictable slip perturbations, walking on a treadmill, and processing vibrotactile cueing. Notably, vibrotactile cueing improved reactive recovery responses compared to the control and post-control trials without further increases in PFC activity.

Previous studies using fNIRS have found increases in PFC activity during balance recovery following external perturbations (e.g., support surface movements or sudden support surface translations) while standing (Mihara et al., 2008; Basso Moro et al., 2014; Fujimoto et al., 2014). The significant increase in bilateral DLPFC, VLPFC, and FPFC activities during the recovery period compared to the standing and walking periods regardless of trial condition (see Figures 5, 6) indicates the involvement of these subregions in reactive recovery responses with or without vibrotactile cueing, which confirms that recovery efforts require more attentional resources than walking or standing. Higher bilateral DLPFC activity for walking than standing, regardless of trial condition, may confirm that walking on the treadmill requires more attentional resources due to the dynamic balance control, as supported by previous findings that walking on a treadmill requires more attention than walking on stable ground (Clark et al., 2014; Mirelman et al., 2014).

Our previous studies have demonstrated that vibrotactile cueing facilitates immediate reactive recovery responses to unpredictable *trip perturbations* that cause the body to move forward (Lee et al., 2016, 2017a), whereas this study demonstrates that vibrotactile cueing facilitates immediate reactive recovery responses to unpredictable *slip perturbations* that cause the body to move backward. During the recovery period, the cueing trial resulted in a lower trunk AD, trunk ROM, and COM ROM, a higher minimum COM position, and a faster step response time compared to the control and post-control trials. Consistent with our previous studies (Lee et al., 2016, 2017a), the results confirm that vibrotactile cueing eliminates the time uncertainty that facilitates the adequate timing of reactive motor responses after a slip perturbation. Following previous studies (Daffner et al., 2000; Pochon et al., 2001), we assume that a precue alerts the PFC to prepare for action by triggering the learned generic slip recovery motor program (Kondo et al., 2017). The kinematic results of the catch trial also indicate that vibrotactile cueing does not trigger anticipatory motor action because the kinematic behavior is similar during the recovery and walking periods (see Figure 8). Therefore, we suggest the following interpretations:

1. Because DLPFC plays a crucial role in attentional tasks, responses to novel events, and preparation for action (Mesulam, 1986; Daffner et al., 2000; Pochon et al., 2001; Momennejad and Haynes, 2012), we attribute the increased bilateral DLPFC activity during the recovery period to the increased need for attentional resources in response to slip perturbations. Unlike overground walking (Clark et al., 2014), increased bilateral DLPFC activity during the walking period compared to the standing period may be associated with the increased need required by balance constraints, such as tight gait speed control to prevent falling while walking on the treadmill. Staring at the “X” mark while walking may be another attention requirement for a secondary task.
2. Because VLPFC plays a crucial role in reflexive reorienting, action updating, motor inhibition, and timing for action (Levy and Wagner, 2011; Momennejad and Haynes, 2012), and from a neurophysiological perspective, it links with the premotor and sensory cortices (Sakagami and Pan, 2007), the increased bilateral VLPFC activity during the recovery period correlates with updating sensory information and recalling and executing the timing of subsequent motor manipulations for stabilizing gait sequences after slip perturbations. Finding no increase in bilateral VLPFC activity during the walking period compared to the standing period confirms this interpretation.
3. Because FPFC plays a crucial role in planning, monitoring, integrating self-generated information, and attention allocation and reallocation (Cummings, 1993; Braver and Bongiolatti, 2002), we interpret that the increase in bilateral FPFC activity during the recovery period compared to the standing and walking periods correlates with planning and integrating the motor responses for stabilizing and executing balance and gait recovery after slip perturbations.
4. Because OFC has a fundamental role in reinforce-related learning, goal-directed action selection, controlling reinforce-related behavior, and emotional processing (Rolls, 2004), it is reasonable to assume that no changes in bilateral OFC demonstrate that repetition has no learning effect. Notably, our previous study demonstrated increased bilateral OFC activity after slip perturbation learning trials (Lee et al., 2020).

We conclude that vibrotactile cueing contributes significantly to preparing the brain and the body for action, action updating, and attention allocation and reallocation, which the response latency results (see Figure 7) and the kinematic results (see Figure 8) confirm. Significantly lower bilateral DLPFC, VLPFC, and FPFC activities for the catch trial compared to the cueing, control, and post-control trials also support our conclusion. The insignificant difference in bilateral DLPFC, VLPFC, and FPFC activities between the cueing trial and the control and post-control trials confirms that vibrotactile cueing does not require more involvement of the PFC subregions following unpredictable slip perturbations, although bilateral DLPFC, VLPFC, and FPFC activities increase in the catch trials. Additionally, the insignificant difference in the average step response time between the cueing and catch trials confirms that vibrotactile cueing causes reflex responses (see Figure 9), which are consistent with the findings of our previous studies (Lee et al., 2016, 2017a).

The main limitations of this study include its relatively small sample size, single participant cohort, and single perturbation magnitude; no period before standing and after recovery (i.e., seated rest); and no measurement of systemic physiological changes. Future research will add measurement devices to record systemic variables (e.g., heart rate, blood pressure, breathing rate, and carbon dioxide concentration) (Tachtsidis and Scholkmann, 2016) and the signal processing method for fNIRS data by incorporating biophysical modeling approaches (e.g., Caldwell et al., 2016) to further reduce physiological confounding effects. Having made these technical improvements, we will study the effects of different perturbation magnitudes on kinematic and kinetic behaviors and PFC activity in balance-impaired individuals, using a larger sample size. Future exploration of the effects of different magnitudes (i.e., different accelerations) of slip perturbations on bilateral DLPFC, VLPFC, and FPFC activities should confirm how vibrotactile cueing can reduce the attentional resources required to activate a reactive recovery response. The additional limitations of this study are the lack of investigation into the PFC and kinematic responses to vibrotactile cueing alerts and the learning effects after multiple exposures to slip perturbations with vibrotactile cueing, which should be explored in our future studies.

## 5. Conclusion

Regardless of the presence of vibrotactile cueing, bilateral DLPFC, VLPFC, and FPFC activities substantially increased during the recovery period following unpredictable slip perturbations. Vibrotactile cueing significantly improved kinematic behavior with no further increases in PFC activity during the recovery period and confirmed the beneficial effects of precues on reactive recovery responses following unpredictable slip perturbations. Without the slip perturbation, vibrotactile cueing also increased bilateral DLPFC, VLPFC, and FPFC activities, but the increased activity was significantly less than the activity in the control, cueing, and post-control trials with and without vibrotactile cueing. While vibrotactile cueing will provide users with navigational guidance to avoid upcoming hazards, longer cueing durations or more complex coding schemes are likely to require more attentional resources for interpretation. We believe that our findings can improve the development of fall reduction and prevention technologies that have vibrotactile cueing alerts.

## Data availability statement

The original contributions presented in the study are included in the article/supplementary material, further inquiries can be directed to the corresponding author.

## Ethics statement

The studies involving humans were approved by Institutional Review Boards of the University of Houston. The studies were conducted in accordance with the local legislation and institutional requirements. The participants provided their written informed consent to participate in this study.

## Author contributions

B-CL conceived the study, designed the experimental protocols, supervised data collection, performed data and statistical analysis, interpreted the results, and drafted the manuscript. JC performed data analysis and drafted the manuscript. JA assisted with data interpretation and manuscript preparation. BM assisted with designing the experimental protocols, interpreted the results, and drafted the manuscript. All authors contributed to the article and approved the submitted version.
